# Relapsing optic neuritis and meningoencephalitis in a child: case report of delayed diagnosis of MOG-IgG syndrome

**DOI:** 10.1186/s12883-019-1324-4

**Published:** 2019-05-09

**Authors:** Xiaonan Zhong, Yanyu Chang, Sha Tan, Jingqi Wang, Xiaobo Sun, Aimin Wu, Lisheng Peng, Alexander Y. Lau, Allan G. Kermode, Wei Qiu

**Affiliations:** 1 Department of Neurology, The Third Affiliated Hospital of Sun Yat-sen University, No.600 Tianhe Road, Guangzhou, 510630 Guangdong Province China; 2 Department of Medicine and Therapeutics, The Chinese University of Hong Kong, Hong Kong Special Administrative Region, China; 3 Centre for Neuromuscular and Neurological Disorders, University of Western Australia, Department of Neurology, Sir Charles Gairdner Hospital, Queen Elizabeth II Medical Centre, Perth, Western Australia Australia; 4 Institute of Immunology and Infectious Diseases, Murdoch University, Perth, Western Australia Australia

**Keywords:** MOG-IgG, Meningoencephalitis, Demyelinating disease

## Abstract

**Background:**

Recurrent optic neuritis (ON) was previously thought to be associated with multiple sclerosis (MS) and neuromyelitis optica spectrum disorders (NMOSD). Meningoencephalitis has recently been suggested to be a clinical finding typical of myelin oligodendrocyte glycoprotein (MOG) encephalomyelitis. We report a Chinese patient with recurrent ON at disease initiation, who had a delayed diagnosis of MOG-IgG syndrome, until recurrent meningoencephalitis appeared and serum MOG-IgG was detected.

**Case presentation:**

From the age of 7 years, an AQP4-IgG negative female patient had 10 disease recurrences, including 4 episodes of recurrent ON, 4 episodes of fever and meningoencephalitis, and 2 episodes of ON as well as meningoencephalitis. She was initially diagnosed as recurrent ON and treated with glucocorticoids followed by gradual tapering when ON reoccurred. Later, she was diagnosed as central nervous system infection when fever and meningoencephalitis appeared, and antiviral drugs and glucocorticoids were used. However, when she returned to our department for follow-up on July 2017, the results of serum demyelinating autoimmune antibody revealed positive MOG-IgG (titer 1:320 by an in-house, cell-based assay using live cells transfected with full-length human MOG). A diagnosis of MOG-IgG syndrome was established.

**Conclusions:**

Testing for MOG-IgG in atypical MS and NMOSD patients, and patients with meningoencephalitis with a history of relapsing demyelinating symptoms is warranted.

**Electronic supplementary material:**

The online version of this article (10.1186/s12883-019-1324-4) contains supplementary material, which is available to authorized users.

## Introduction

Recurrent optic neuritis (ON) was previously thought to be associated with other idiopathic inflammatory demyelinating disease, such as multiple sclerosis (MS) and neuromyelitis optica spectrum disorders (NMOSD) [1, 2]. Currently, most neurologists realize that it can also be a symptom typical of MOG-IgG syndrome. However, MOG-IgG syndrome may be associated with a wide spectrum of symptoms. Of note, meningoencephalitis was recently reported in a MOG-IgG syndrome case [3]. We report a Chinese patient with recurrent ON at disease initiation, who had a delayed diagnosis of MOG-IgG syndrome until recurrent meningoencephalitis appeared and serum MOG-IgG was detected.

## Case report

**Fig. 1:**
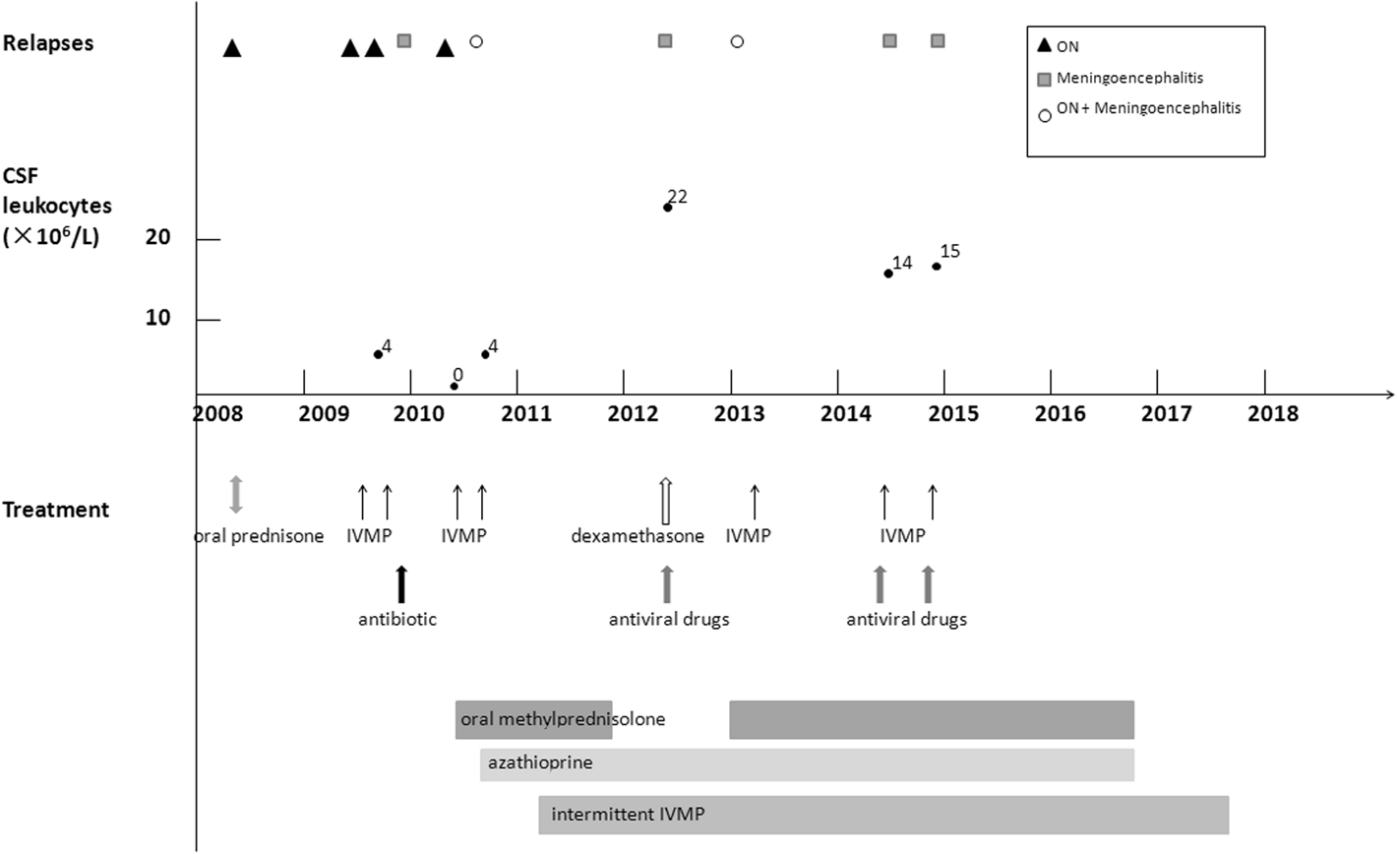
Clinical symptoms, MRI, CSF leukocytes and treatment since the onset of the disease. From the age of 7 years, a female patient had 10 disease recurrences, including 4 episodes of recurrent optic neuritis, 4 episodes of fever and meningoencephalitis, and 2 episodes of optic neuritis as well as meningoencephalitis. High dose intravenous methylprednisolone was the main treatment for relapses. Azathioprine, oral methylprednisolone and intermittent intravenous methylprednisolone were used in remission

From the age of 7 years (March 2008), a female patient had 10 disease recurrences, including 4 episodes of recurrent ON, 4 episodes of fever and meningoencephalitis, and 2 episodes of ON as well as meningoencephalitis (Fig. 1).

She had 4 episodes of recurrent ON. She first presented with rapid visual loss in both eyes and no light perception within 3 days after developing a cold at 7 years old. The responsible lesions were identified in the bilateral optic nerve on MRI, and her brain MRI was normal. At the age of 8 years old, she developed severe worsening of vision to 0.1 in the right eye. Three months later, she suffered a decrease of left-eye vision (0.1) and numbness in both lower extremities. When she came to our hospital, physical examination revealed a left-eye vision of 0.6, right-eye vision of 1.0 and a marked sensory level at T5. Rheumatic autoantibodies and microorganism (Toxoplasma, Rubella Virus, Cytomegalovirus, Herpes Simplex Virus, Epstein-Barr Virus) antibodies screening were negative. Detection of AQP4-IgG in the serum and cerebrospinal fluid (CSF) using aquaporin-4-transfected cells from a commercial sampling kit (Euroimmun, Germany) was negative. CSF analysis demonstrated normal cell counts and biochemistry, and a lack of oligoclonal bands (OCB). Whole spinal cord MRI showed no lesions. When she was 9 years old, the patient was hospitalized with complaints of impaired vision in both eyes and numbness in her left lower extremity. Physical examination revealed a visual acuity of 0.1 in both eyes and decreased sensation below T5. AQP4-IgG was not detected in her serum and CSF. Her CSF examination was normal. Cerebral MRI revealed multiple small, hyperintense T2-weighted lesions in the bilateral frontal lobes. Repeat MRI of the spinal cord showed no lesions. During these relapses, she was diagnosed as recurrent ON and treated with glucocorticoids followed by gradual tapering, and her vision eventually returned to normal.

**Fig. 2:**
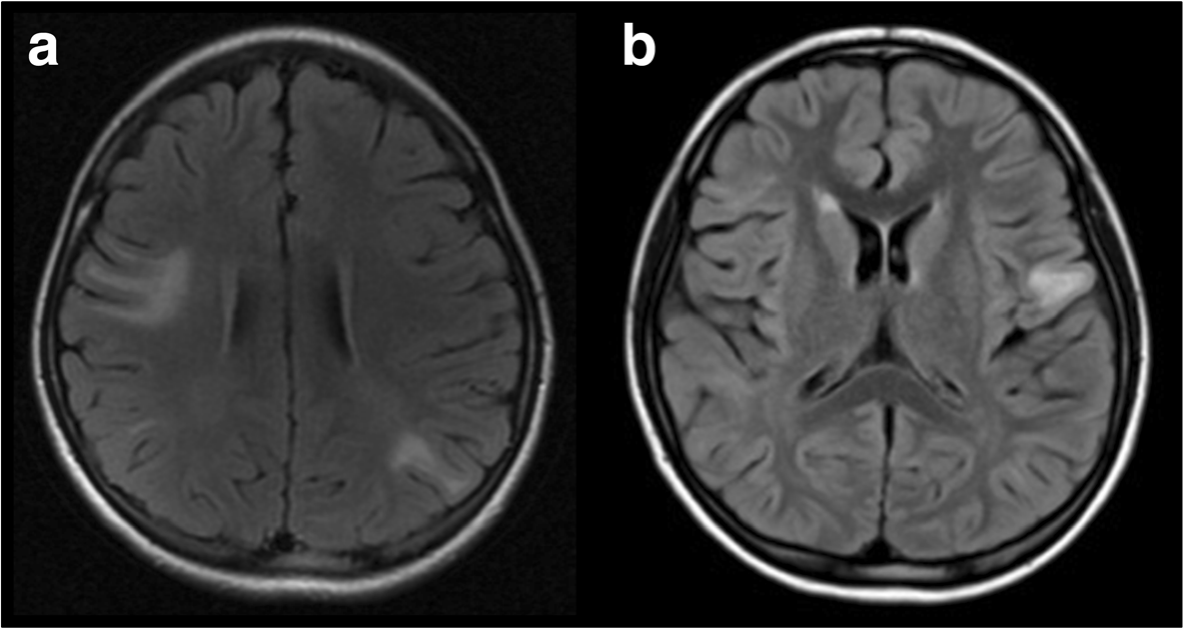
Cortex and subcortical lesions on Brain MRI. **a** Brain MRI on January 23, 2013: lesions in bilateral frontal lobe, left parietal lobe and right temporal lobe were seen, cortex and subcortical can be found in right frontal lobe and left parietal lobe; **b** Brain MRI on May 27, 2014: new lesions were observed in left frontal lobe

She also had 4 relapses of fever and meningoencephalitis. Also at 8 years old, the patient had sustained fever (maximum body temperature 39.4 °C), headache, nausea, vomiting and was lethargic. At the age of 11, fever (maximum body temperature 38.1 °C), headache, nausea, vomiting and lethargy appeared again. Neurological examination indicated positive pyramidal signs of both lower extremities. Her CSF leukocyte level was 22.0 × 10^6^/L. On brain MRI, lesions in the bilateral frontal lobe and right temporal lobe grew larger than before. When 13 years old, the patient developed psychiatric symptoms, including sluggishness, providing irrelevant answers to questions, intermittent speech, declining memory, and fever (maximum body temperature 38.3 °C). Then, headache, nausea, and vomiting followed. Increase leukocyte levels were found in the CSF (14.0 × 10^6^/L). New lesions were observed in the left frontal lobe and temporal lobe on brain MRI (Fig. 2-b). Her electroencephalogram was abnormal with rhythm slowing, and a θ wave was noted. At the age of 14 years old, headache, nausea, vomiting and lethargy appeared again. The CSF leukocyte level was 15.0 × 10^6^/L. The result of microbial (fungi, bacteria and DNA viruses) second generation sequencing was negative. When the meningoencephalitis symptoms appeared, she was diagnosed as upper respiratory tract infection or viral meningitis, and was treated with antibiotic/antiviral drugs and glucocorticoids. Subsequently, her neurological symptoms gradually improved.

In another 2 relapses, she suffered ON and meningoencephalitis. At 9 years old, she had a headache, followed by fever (maximum body temperature 38.6 °C), nausea, vomiting and lethargy. Antibiotics were administered for upper respiratory tract infection at a regional hospital, but the above symptoms did not improve. Five days later, she suffered visual disturbances in both eyes. Physical examination showed a left-eye vision of 0.6, right-eye vision of 0.25 and sensory level at T5. CSF leukocytes were elevated slightly (4.0 × 10^6^/L). Although asymptomatic, new lesions in the bilateral frontal, right temporal, parietal, and insular lobes were observed. At 11 years old, she experienced headache, and suffered sudden visual loss in both eyes 10 days later. Physical examination revealed a visual acuity of 0.3 in the left eye, 0.5 in the right eye, and a marked sensory level at T4. On brain MRI, lesions were increased in the bilateral frontal, left parietal, and right temporal lobes (Fig. 2**-a**). In these two recurrences, her symptoms, including meningoencephalitis, responded well to intravenous methylprednisolone (IVMP) therapy and oral tapering.

Since the fifth relapse in April 2010, low-dose oral methylprednisolone maintenance therapy, azathioprine and intermittent IVMP therapy (once every 3 months) were successively used during remission. On October 2016, the patient stopped taking methylprednisolone and azathioprine by herself. She was stable and intermittent IVMP was stopped in July 2017 (Fig. 1). However, when she returned to our department for follow-up on July 2017, the results of serum demyelinating autoimmune antibody analysis was positive MOG-IgG (titer 1:320 by in-house, cell-based assay using live cells transfected with full-length human MOG [4]) and negative AQP4-IgG.

At the latest follow-up in July 2018, her condition remained stable. Her conventional brain MRI was similar to 1 year earlier. Multimodal MRI suggests that lesions are consistent with imaging characteristics of typical demyelinating disease (Additional file 1) and a lesion at C6/7 was found on spinal MRI (Additional file 2). Repeat serum demyelinating autoimmune antibody testing revealed positive MOG-IgG (titer 1:320) and negative AQP4-IgG in this case.

## Discussion

In this case report a MOG-IgG syndrome patient with recurrent ON, clinical symptoms consistent with myelitis with a sensory level, and recurrent meningoencephalitis was described.

At disease initiation, the patient suffered from recurrent ON, which is common in MS and NMOSD. For this female patient, an acute onset, a recurrent disease course, and the treatable effect with immunomodulatory therapy suggested her ON was associated with a chronic inflammatory demyelinating disease. As for MS, the lesion location of our patient was not consistent with the characteristic MS lesions located in the periventricular region; meantime, our patient’s OCB which is a characteristic biomarker for MS was negative. When it comes to NMOSD, the serum AQP4-IgG of our patient was repeatedly negative. On the basis of the diagnostic criteria for NMOSD without AQP4-IgG, the patient should have at least two typical core clinical characteristics. Though our patient had recurrent ON, she did not have another characteristic core symptom. Therefore a diagnosis of MS or NMOSD could not be established according to the current McDonald and Wingerchuk criteria respectively. Thus, the diagnosis of idiopathic recurrent ON was made after the first few years of close follow-up. However, recurrent ON is also a typical symptom of MOG-IgG syndrome.

Our patient also had other symptoms atypical for MS and NMOSD, including repeated fever, headache, and nausea/vomiting without an MRI lesion in the area postrema. Though our patient did not have clinical manifestations of infection such as upper respiratory tract or gastrointestinal symptoms, isolated fever may still easily be mistaken for precursor infection and may often be ignored. Headache, nausea, and vomiting accompanied by elevated CSF leukocytes suggest meningoencephalitis, which has recently been described in MOG-IgG syndrome [3], but they are rarely reported in MS. Although nausea/vomiting is the manifestation of area postrema syndrome in NMOSD, this core clinical characteristic requires a lesion in the area postrema, and therefore nausea/vomiting without a lesion in area postrema could not be explained by NMOSD. Another finding was cortical and subcortical lesions on brain MRI which may explain the meningoencephalitic symptoms of the patient. The presence of fever, meningoencephalitic symptoms, abnormal CSF presentation and atypical lesions on MRI led to a misdiagnosis of central nervous system infection in our patient. However, repeated meningoencephalitis prompted us to consider diagnoses other than central nervous system infection. We detected a sustained high titer of serum MOG-IgG leading to a diagnosis of MOG-IgG syndrome.

Acute disseminated encephalomyelitis (ADEM) should also be initially suspected, but our patient lacked of some characteristic symptoms of ADEM. ADEM is a syndrome classically with onset after infection or vaccination. However, our patient had sustained fever during her attack without any prodromal viral illness or vaccination, and that was one reason why meningoencephalitis seemed more reasonable than ADEM to the neurologists. Moreover, the typical MRI lesions of ADEM are diffuse, poorly demarcated, symmetrical large white matter lesions, while our patient had asymmetric cortical and subcortical lesions. Furthermore, our patient also had a highly active and relapsing recurrent condition with 10 attacks. In contrast by definition ADEM has a low relapse frequency [5]. Therefore, relapsing disease following ADEM that occurs beyond a second encephalopathic event is no longer consistent with multiphasic ADEM but rather indicates a chronic disorder [5]. It is also worth noting that in the latest ADEM criteria by International Pediatric Multiple Sclerosis Study Group, ADEM is a heterogeneous entity and is best viewed as a syndrome rather than a specific disorder [5]. For our patient, MOG-IgG syndrome better explains the recurrent clinical symptoms and MRI findings, as opposed to an extremely atypical recurrent ADEM syndrome comprising 10 documented relapses over several years. Many manifestations of the MOG-IgG syndrome which are rarely seen in ADEM were observed in my patient, such as meningoencephalitis symptoms including fever, headache, nausea and vomiting [5].

MOG-IgG syndrome has symptoms similar to other idiopathic inflammatory demyelinating diseases, and may easily be confused with them. Meningoencephalitis may be helpful for the differential diagnosis and testing for MOG-IgG in atypical MS and NMOSD patients can be useful. On the other hand, patients with meningoencephalitis are often diagnosed as central nervous system infection. Our findings suggest that autoimmune conditions, including MOG-IgG syndrome, should be considered in patients with meningoencephalitis.

## Conclusion

Testing for MOG-IgG in atypical MS and NMOSD patients, and patients with meningoencephalitis with a history of relapsing demyelinating symptoms is warranted.

## Additional files

Additional file 1: Multimode brain MRI. (A) On the magnetic resonance spectroscopy (MRS) sequence at July 2017, increase in choline compounds (Cho) was found in the right frontal lobe lesion; (B) MRS sequence was performed again at July 2018; lesions were with Cho elevated and had a decline in N-acetyl-aspartate (NAA); (C,D) Small veins pass through the center of the lesion can be seen on the susceptibility weighted imaging (SWI); (E) No abnormalities were found in magnetic resonance angiography and magnetic resonance venogram. (TIF 4860 kb)

Additional file 2: Spinal cord MRI. (A) Spinal cord MRI was normal at August 2010; (B) On July 2018, some prominence in the central canal in spinal cord was found at vertebral body of C6/7. (TIF 4320 kb)

## Abbreviations

AQP4: Aquaporin-4
CSF: Cerebrospinal fluid
IVMP: Intravenous methylprednisolone
MOG: Myelin oligodendrocyte glycoprotein
MOG-IgG: Immunoglobulin G against myelin oligodendrocyte glycoprotein
MRI: Magnetic resonance imaging
MS: Multiple sclerosis
NMOSD: Neuromyelitis optica spectrum disorders
OCB: Oligoclonal bands
ON: Optic neuritis
