# Supplementary figures and images for: Relapsing optic neuritis and meningoencephalitis in a child: case report of delayed diagnosis of MOG-IgG syndrome
Source: BMC Neurol. 2019 May 9;19:94. doi: 10.1186/s12883-019-1324-4 (PMC6506947; doi:10.1186/s12883-019-1324-4)

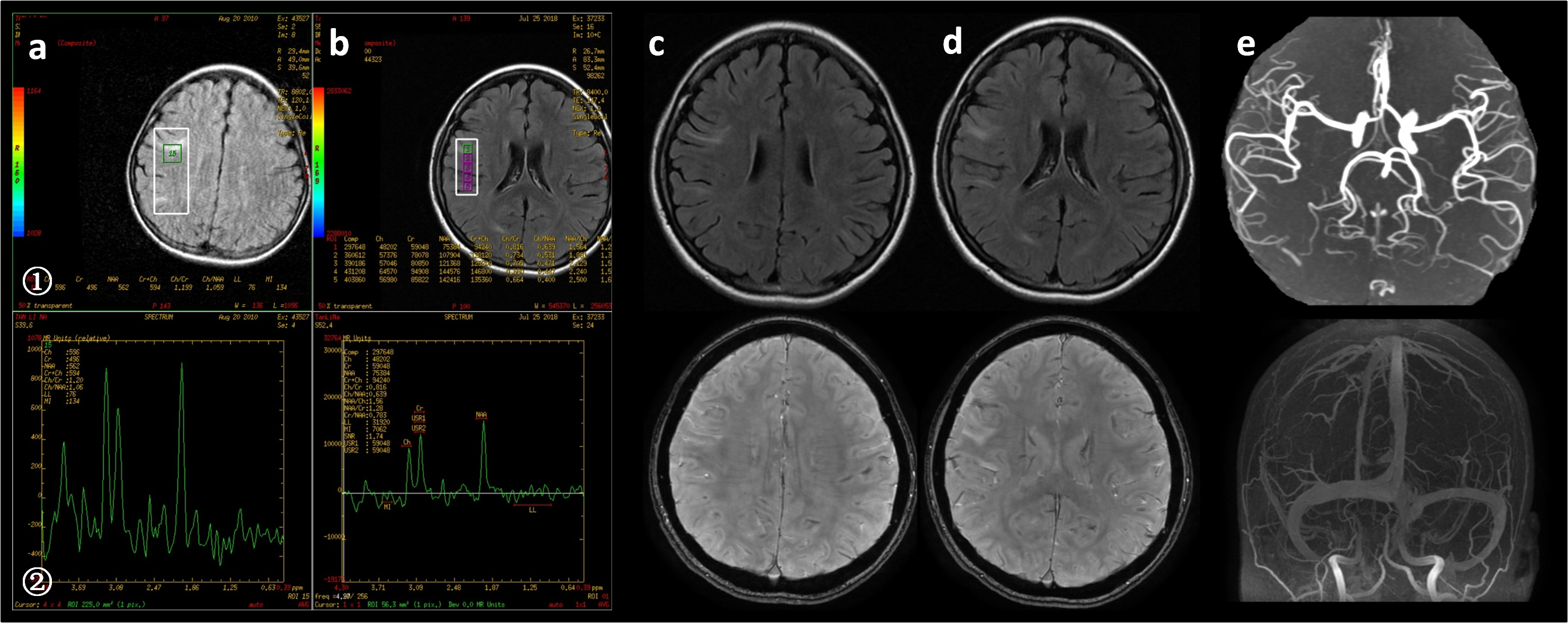

Supplement: Supplementary file 1 — Multimode brain MRI. (A) On the magnetic resonance spectroscopy (MRS) sequence at July 2017, increase in choline compounds (Cho) was found in the right frontal lobe lesion; (B) MRS sequence was performed again at July 2018; lesions were with Cho elevated and had a decline in N-acetyl-aspartate (NAA); (C,D) Small veins pass through the center of the lesion can be seen on the susceptibility weighted imaging (SWI); (E) No abnormalities were found in magnetic resonance angiography and magnetic resonance venogram. (TIF 4860 kb) [file 12883_2019_1324_MOESM1_ESM.tif]

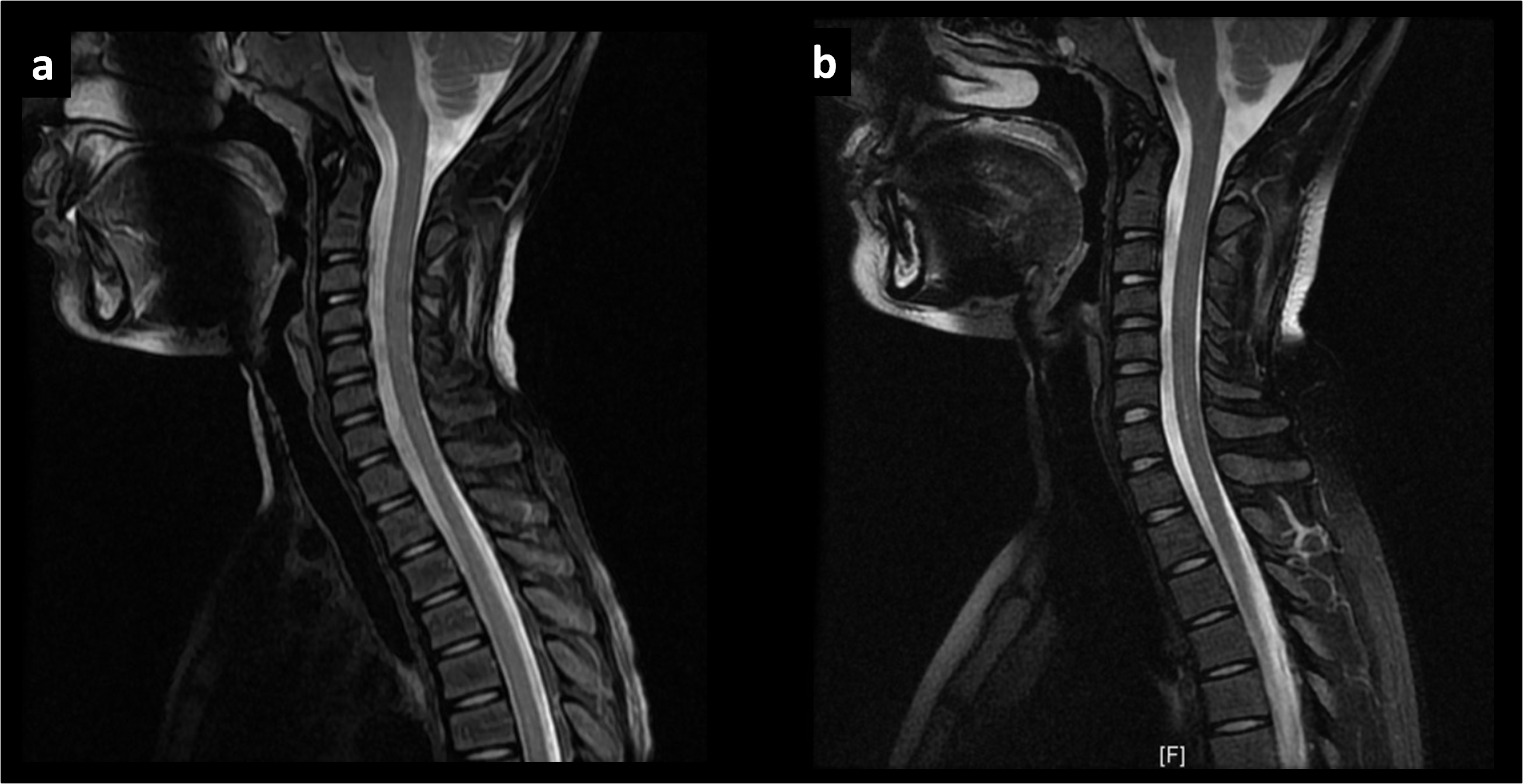

Supplement: Supplementary file 2 — Spinal cord MRI. (A) Spinal cord MRI was normal at August 2010; (B) On July 2018, some prominence in the central canal in spinal cord was found at vertebral body of C6/7. (TIF 4320 kb) [file 12883_2019_1324_MOESM2_ESM.tif]
